# Characterization of the rotavirus assembly pathway *in situ* using cryoelectron tomography

**DOI:** 10.1016/j.chom.2023.03.004

**Published:** 2023-03-29

**Authors:** Pranav N.M. Shah, James B. Gilchrist, Björn O. Forsberg, Alister Burt, Andrew Howe, Shyamal Mosalaganti, William Wan, Julika Radecke, Yuriy Chaban, Geoff Sutton, David I. Stuart, Mark Boyce

**Affiliations:** 1 Division of Structural Biology, Nuffield Department of Medicine, University of Oxford, The Wellcome Centre for Human Genetics, Headington, Oxford, UK; 2 CAMS Oxford Institute, Nuffield Department of Medicine, University of Oxford, Old Road Campus, Headington, Oxford, UK; 3 Diamond Light Source Ltd, Harwell Science & Innovation Campus, Didcot, UK; 4 Department of Physiology and Pharmacology, Karolinska Institute, Stockholm, Sweden; 5 Laboratory of Molecular Biology, Cambridge Biomedical Campus, Francis Crick Avenue, Cambridge, UK; 6 Life Sciences Institute and Department of Cell and Developmental Biology, University of Michigan, Ann Arbor, MI, USA; 7 Vanderbilt University Center for Structural Biology, PMB 407917, 465 21st Ave S, 5140 MRB3, Nashville, TN, USA

## Abstract

Rotavirus assembly is a complex process that involves the stepwise acquisition of protein layers in distinct intracellular locations to form the fully assembled particle. Understanding and visualization of the assembly process has been hampered by the inaccessibility of unstable intermediates. We characterize the assembly pathway of group A rotaviruses observed *in situ* within cryo-preserved infected cells through the use of cryoelectron tomography of cellular lamellae. Our findings demonstrate that the viral polymerase VP1 recruits viral genomes during particle assembly, as revealed by infecting with a conditionally lethal mutant. Additionally, pharmacological inhibition to arrest the transiently enveloped stage uncovered a unique conformation of the VP4 spike. Subtomogram averaging provided atomic models of four intermediate states, including a pre-packaging single-layered intermediate, the double-layered particle, the transiently enveloped double-layered particle, and the fully assembled triple-layered virus particle. In summary, these complementary approaches enable us to elucidate the discrete steps involved in forming an intracellular rotavirus particle.

## Graphical abstract

**Graphical abstract:**
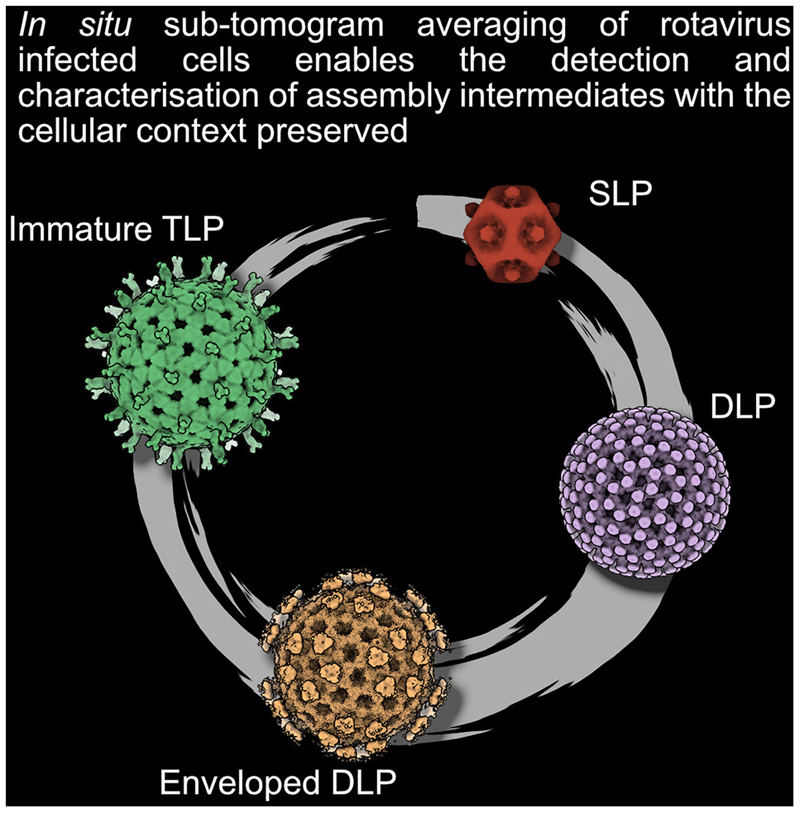

## Introduction

Members of the family *Reoviridae* include pathogens of human and veterinary importance, and rotaviruses are a major cause of infant mortality due to viral gastroenteritis.^1^ The viral particles are characterized by their multi-layered icosahedral shells that enclose a segmented, double-stranded RNA genome. The family is divided structurally and taxonomically into turreted and non-turreted members based on the location of the capping enzyme, situated either on the outer surface of the inner protein layer or inside the virion. The structural features of the outer layers of the capsid in *Reoviridae* vary, with only the inner layer being structurally well conserved throughout the family.

**Figure 1:**
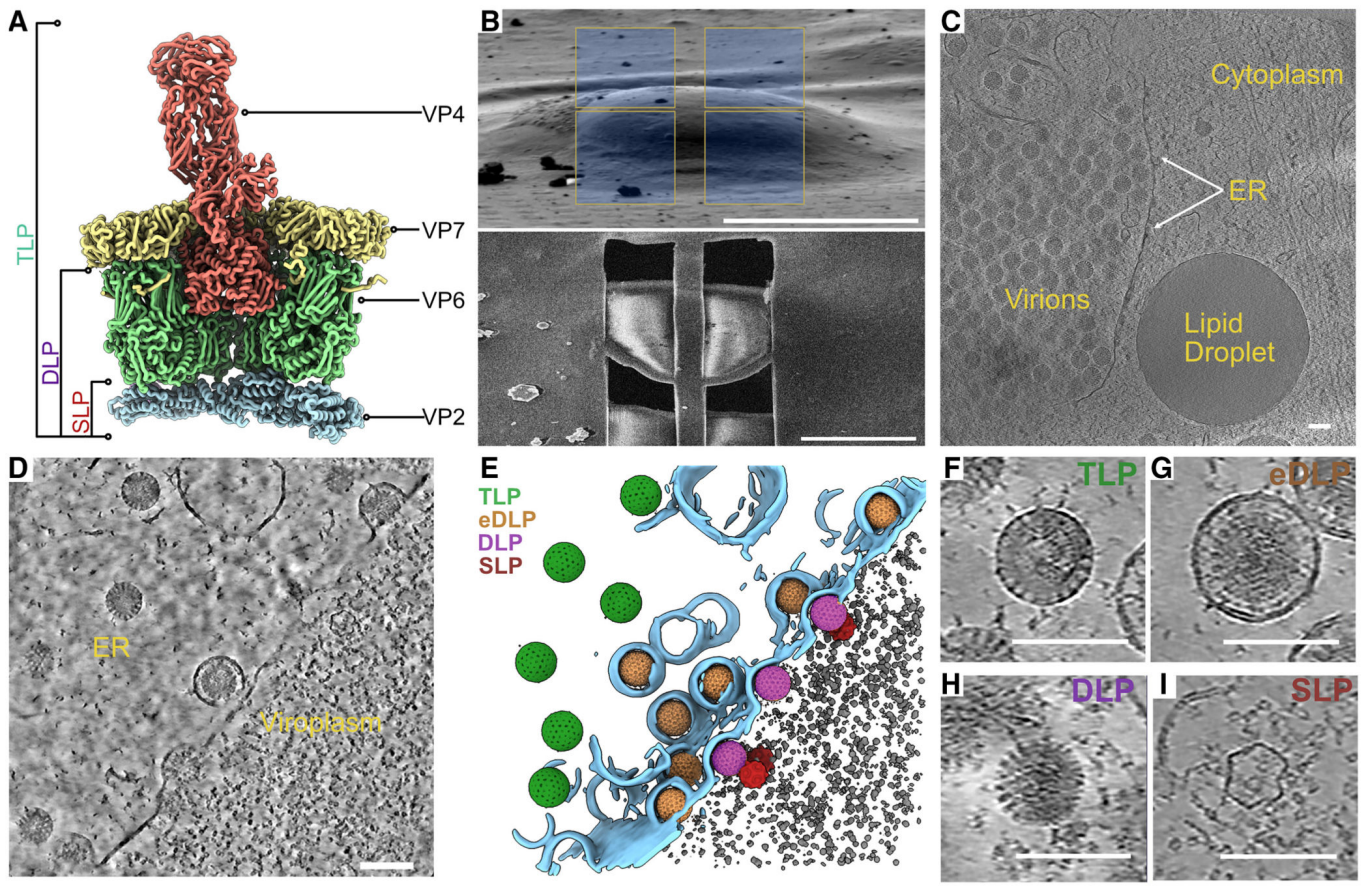
Cryo-FIB milling of rotavirus-infected cells reveals virus assembly intermediates (A) A cutaway view of a portion of the rotavirus capsid generated from this study is depicted as a liquorice cartoon. Protein subunits present in the virion are VP2 (light blue), VP6 (light green), VP7 (light yellow), and VP4 (salmon). The subunits that constitute the different assembly intermediates are indicated. (B) SEM image of a frozen vitrified MA104 cell infected with rotavirus prior to milling with milling windows indicated and viewed from above after milling. Scale bars, 15 μm. (C) Low-magnification overview of an infected cell with clusters of fully assembled virions in the ER. Scale bar, 100 nm. (D) Computational slice through a tomogram of an infected cell. The tomogram contrast has been enhanced using a deep learning-based denoising routine (STAR Methods). Scale bar, 100 nm. (E) Segmentation of the tomogram in (D) with virus particles in various stages of assembly. Color scheme indicates the different particle types TLP (green), eDLP (orange), DLP (purple), and SLP (red). (F–I) Close-up views of rotavirus assembly intermediates. Scale bars, 100 nm.

Rotavirus, a non-turreted member of the *Reoviridae*, has a triple-layered capsid that is assembled stepwise in two separate compartments in the infected cell: the virus-induced viroplasm and the endoplasmic reticulum (ER). Rotavirus virions contain 11 unique double-stranded RNA (dsRNA) genome segments and the enzymatic apparatus to synthesize transcripts from them following infection of the host cell. The icosahedral inner layer of the virus, composed of 120 copies of VP2 (Figure 1A), is in direct contact with the dsRNA genome and encloses 11 copies of the viral polymerase VP1 and a similar but undetermined number of copies of the capping enzyme VP3.^2^ The intermediate layer consists of 260 homotrimers of VP6 arranged in a T = 13 lattice on the surface of the inner layer (Figure 1A). The outermost protein layer consists of 260 trimers of the glycosylated protein VP7, with the inter-subunit interfaces stabilized by Ca^2+^ ions (Figure 1A)^2,3^ and 60 trimers of VP4 (Figure 1A) forming spikes which mediate virus attachment and entry.^2,4^ The particle with all three protein shells present is known as a triple-layered particle (TLP) and is the fully assembled form of the virus (Figure 1A).^2^

Trypsin-like proteases present in the intestinal lumen render the TLP competent for entry by cleaving VP4 spikes at specific sites into two fragments, VP5* and VP8*.^5^ Virus entry occurs when the TLP binds to ganglioside receptors on the surface of the cell^4^ and initiates uptake through membrane invagination and budding.^6^ During the entry process the cleaved trimeric VP4 spontaneously undergoes an extensive rearrangement, from an asymmetric conformation to a reversed, symmetric conformation.^7^ C-terminal VP4 helical domains extend inward in the TLP and are buried in the middle, VP6, layer. The conformational changes on entry cause these to become exposed and to interact with the limiting membrane of the vesicle, presumably leading to membrane perforation.^7^ The loss of Ca^2+^ ions from the incoming virus-containing vesicle causes the VP7 layer to disassemble and the transcriptionally active double-layered particle (DLP) is released into the cytoplasm.^8,9^

Much of the subsequent replication cycle and assembly pathway, including genome assortment, assembly up to the formation of DLPs and secondary transcription, occurs in structures that assemble in the cytoplasm of infected cells termed viro-plasms.^1,10,11^ Rotavirus viroplasms are apposed to the ER, contain two non-structural proteins, NSP2 and NSP5,^10,11^ and are the site where interactions between viral structural proteins VP1, VP2, VP3, and VP6 drive particle assembly, as well as the packaging of the single-stranded form of viral RNA genome segments into the assembling particle.^10^ The formation of a single-layered particle (SLP) has been inferred as a necessary first step in assembly but has not been observed *in situ*, with the DLP formed following the addition of the intermediate layer protein VP6 being the earliest assembly stage observed in viroplasms.

Following assembly the DLP leaves the viroplasm and although the molecular interactions are not fully characterized, the VP6 layer is known to then mediate interaction with the cytosolic domain of the viral NSP4 protein embedded in the ER membrane.^12,13^ The VP4 spike protein interacts with both NSP4 and VP6, accumulating at the narrow gap between the viroplasm and ER and becoming incorporated into the DLP as it buds through the ER membrane.^12^ The resulting transiently enveloped DLP (eDLP) is released into the ER lumen, where the outer layer protein VP7 is located.^14,15^ Here, the completion of the assembly process occurs through a poorly understood mechanism that leads to the loss of the transient envelope from the eDLP and the addition of VP7 trimers to form the outer layer of the TLP.

Our knowledge of the assembly pathway of rotavirus remains incomplete. For instance, little is known about the early stages of assembly preceding the formation of DLPs, the transiently enveloped eDLP stage, and how VP4 of immature intracellular TLPs differs from the mature infectious extracellular form. In this study we use cryo-focused ion beam (cryo-FIB) milling and cryoelectron tomography (cryo-ET) in combination with a temperature-sensitive mutant and inhibition of ER Ca^2+^ regulation to identify and characterize four distinct assembly intermediates *in situ*. Subtomogram averaging (STA) enables a detailed description of the intracellular TLP form of the virus prior to release and locates the third copy of the lectin-like head domain of VP4 in the asymmetric trimer. To investigate the transiently enveloped eDLP, we analyze infected cells treated with thapsigargin to blockade assembly at this stage, allowing the discovery of an undescribed conformation of VP4 and the mechanism by which the eDLP is tethered to the membrane. Furthermore, we uncover genome-less SLPs inside the viroplasms, indented at the 5-fold similar to those observed with reovirus.^16^ Finally, using a temperature-sensitive mutant, we show that the RNA-dependent RNA polymerase, VP1, has a role in the recruitment of the viral genome into this genome-less SLP.

## Results

### FIB milling of rotavirus-infected cells reveals intermediates of assembly

In an earlier *in situ* cryo-ET study of reovirus (also a member of the *Reoviridae*), we identified and characterized two discrete stages in the assembly pathway; the genome-less SLPs and the completed virions.^16^ Since the rotavirus capsid has an extra protein layer, and the assembly involves the ER, its assembly and maturation pathway would be expected to be more complex. To characterize the assembly pathway, MA104 cells grown on gold grids were infected with rotavirus SA11 at an MOI of 10 and plunge-frozen in liquid ethane at 13 h post-infection (hpi) (STAR Methods). Grids with appropriate ice thickness, as judged by cryo-transmission electron microscopy (cryo-TEM), were further examined with a cryo-scanning electron microscopy (cryo-SEM), and candidate cells were identified (Figure 1B). Next, lamellae were obtained by cryo-FIB milling the cytoplasm adjacent to the nucleus in order to capture the ER with high probability. In total, 11 lamellae were obtained from 2 grids that enabled the acquisition of 85 tilt-series (TS) (STAR Methods). The average thickness of the lamellae was 210 nm (±50 nm).

Low-magnification surveys of the lamellae revealed the typical hallmarks of rotavirus infection, with progeny virions captured in various states of assembly within the cytoplasm, and a lack of involvement of the nucleus (Figures 1C–1E). The ER was extensively reorganized, characterized by gross dilation of the lumen, which contained clusters of TLPs (Figure 1C). As expected, the viroplasms contained DLPs (Figures 1D, 1E, and 1H), but also relatively electron translucent SLPs (Figures 1D, 1E, and 1I). Moreover, DLPs were observed in the process of budding through the ER membrane from the adjacent viroplasm with completely budded eDLPs seen in the lumen (Figures 1D, 1E, and 1G).

Overall, the dataset enabled the identification of 4 distinct intermediates in the assembly pathway; the TLP, the eDLP, the DLP, and the SLP (Figures 1D–1I) of which the eDLP (Figure 1G) and the SLP (Figure 1I) have been, to date, poorly characterized in both their composition and structure, while the DLP and TLP have been characterized, but only in a purified form. Three of the four intermediate forms were sufficiently abundant to yield sub-nanometer maps after STA (see below).

### High-resolution structures of the virus assembly intermediates using STA

**Figure 2:**
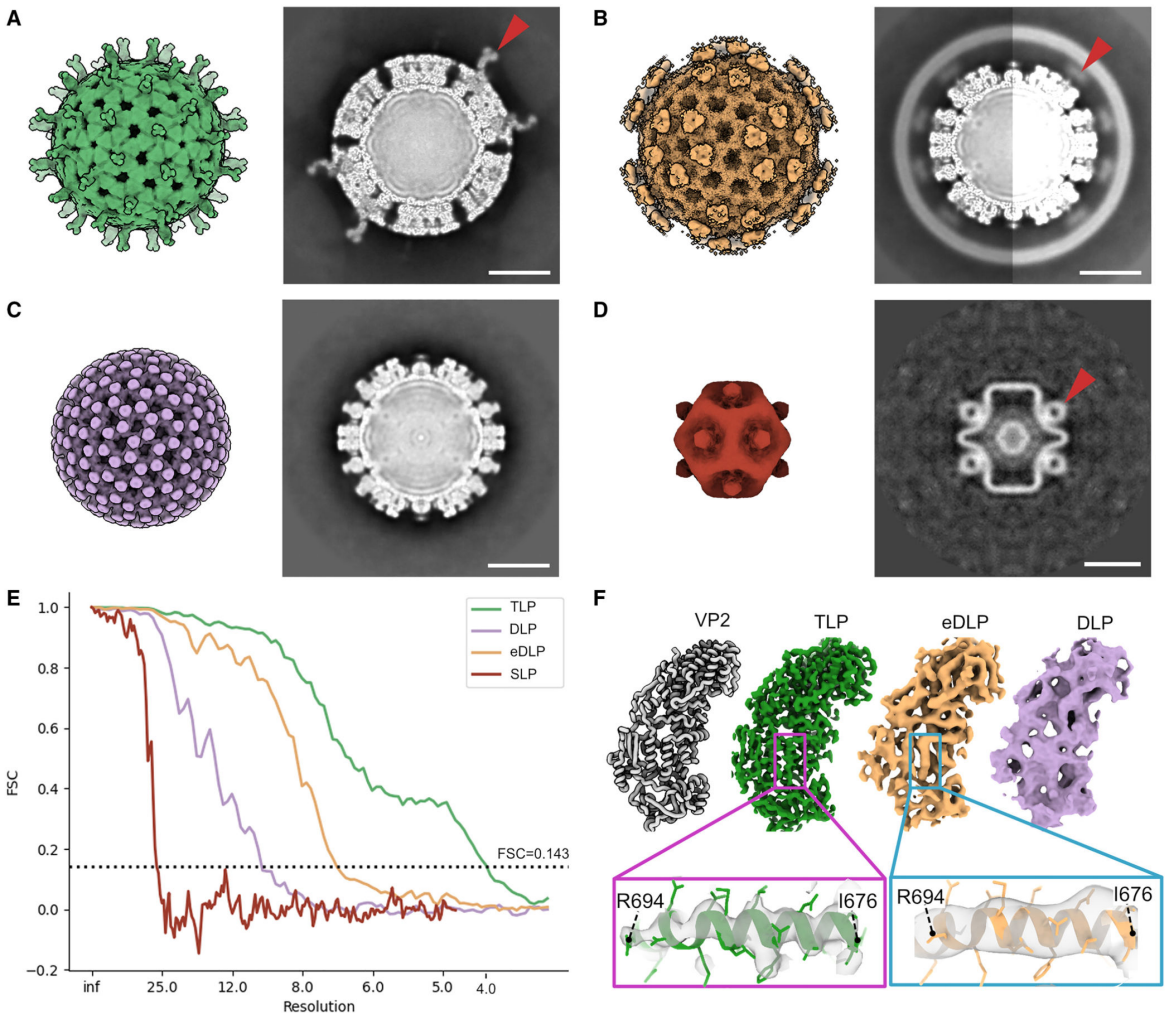
High-resolution subtomogram averages of rotavirus assembly intermediates (A) TLP isosurface. Arrow indicates VP4 in its upright conformation. (B) eDLP isosurface with the membrane masked out. Arrow indicates the connection between the capsid and the membrane. (C) DLP isosurface. (D) SLP isosurface. Arrow indicates pronounced densities present at the 5-fold. (E) FSC traces for pentons of TLP (green), eDLP (orange), DLP (purple), and whole SLP (red). (F) Fit of the inner layer VP2 monomer (gray) in the maps obtained following 5-fold averaging of the pentons. Residues ranging from Arg694 to Ile676 are highlighted in the inset. All scale bars show 25 nm.

From the three-dimensional tomograms (STAR Methods) particles were extracted for the completed TLP (n = 279) (Figure 2A), eDLP (n = 138) (Figure 2B), DLP (n = 42) (Figure 2C), and the SLP (n = 28) (Figure 2D). Each particle type was subjected to subtomogram refinement and averaging within the Warp-Relion-M framework while imposing icosahedral symmetry,^17^ resulting in reconstructions at estimated resolutions of 6.9 Å (TLP), 7.7 Å (eDLP), 12.0 Å (DLP), and 26.9 Å (SLP) (Figures S1A and S1B; Table S2). To improve the resolution, deviations from the icosahedral geometry were permitted by enforcing only 5-fold symmetry, at the icosahedral 5-fold axes, applied to the appropriate asymmetric portion of the capsid. This produced global resolutions of 4.2 Å (TLP), 7.2 Å (eDLP), and 10.2 Å (DLP) (Figures 2E and 2F; Table S2). The SLP could not be refined in this way due to the low number of particles in the dataset. Nevertheless, the SLP density map was found to be remarkably similar to that described for the more abundant reovirus SLP^16^ (see below).

The capsid densities of the TLPs and DLPs observed *in situ* were mostly very similar to those of the corresponding gradient purified particles^2,18^ (Figure 2F, average RMSD for the main chain for TLP, eDLP, and DLP are 0.54, 0.63, and 0.66 Å, respectively). However, significant deviations from these models were identified in the *in situ* TLP and eDLP intermediates (see below).

### *In situ* STA of intracellular TLPs enables the characterization of VP4 prior to exposure to trypsin

**Figure 3:**
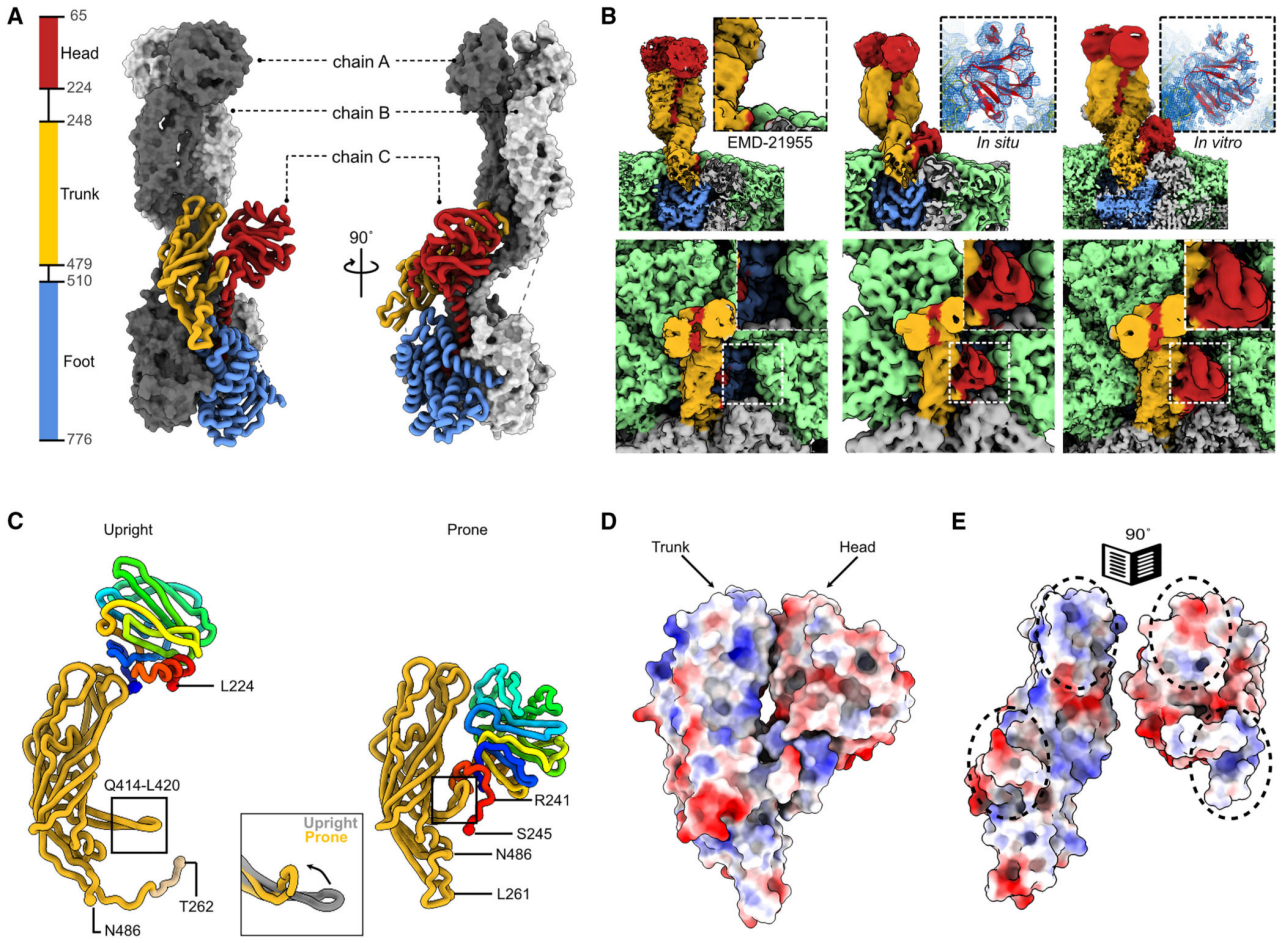
Localization of the VP4-C head domain on the TLP (A) Architecture of the VP4 spike. Chains A and B are represented as surfaces (gray) and chain C as colored liquorice. Foot domain, blue; trunk domain, gold; head domain, red. Residue ranges of the domains are indicated. (B) Isosurface renderings ofVP4 maps. Top row, side views of EMD-21955 (left), *in situ* TLP (middle) and gradient purified TLP (right). Bottom row, top views. VP4 domains are colored as in (A), and the outer capsid layer VP7 is colored green. Insets highlight position of the head domain. (C) Comparison of the upright VP4-A chain with prone VP4-C. The head domain, represented as a Jones rainbow, is rotated by ~117°. The trunk domain is colored yellow. The z axis is perpendicular to the viewing direction. Inset, a least squares superposition of the trunk domains comparing VP4-A (gray) and VP4-C (yellow). (D) Electrostatic potential distribution on the surface of VP4-C. (E) As (D) except that the head and trunk domains are each rotated like an open book. Charge complementation between the two domains is indicated with dotted ovals.

VP4 is a homotrimer of three chains (A–C) associated in an unusual asymmetric arrangement in the infection competent TLP form (Figure 3A). Chains A and B project outward from the particle as a dimer while chain C lies prone (Figure 3A). Each chain consists of three distinct domains; head (residues 65–224), trunk (residues 248–479), and foot (residues 510–776), which are connected via flexible linkers (Figure 3A). Previous atomic structures of rotavirus TLPs were derived from virions exposed to trypsin.^2,7^ This increases their infectivity^19,20^ via an ordered cleavage of VP4 in a trypsin-sensitive region at Arg^231^, Arg^241^, and Arg^247^.^21^ In the mature infectious particle these cleavages generate a small N-terminal fragment VP8* and a large C-terminal fragment, VP5*, which remain non-covalently associated. Two of the three copies of VP8* (VP8*-AB) were localized in high-resolution maps while VP8*-C was not observed (Figure 3B, left). Previous attempts to study trypsin-unexposed virions showed that an asymmetric structure could be formed prior to cleavage but failed to reveal the structure at high resolution.^22^ The intracellular TLP visualized here provides the opportunity to study the structure of VP4 in fully assembled virions prior to release into the trypsin containing extracellular medium.

In our high-resolution icosahedral reconstructions of the *in situ* TLP capsid (Figures 2A and 2E), the VP4 component was poorly resolved due to symmetry-imposed smearing of the density. To improve the resolution, the icosahedral orientation parameters calculated in the previous step were used to extract all potentially spike-containing vertices (n = 16,750). Local refinement was performed to permit deviations from the icosahedral capsid followed by classification without alignment to eliminate low quality subtomograms (54.87%), as judged by the lack of secondary structure detail in the 3D class averages, as well as unoccupied vertices (9%). Classes of subtomograms that exhibited strong secondary structure features were further refined in Relion followed by M, generating a final map of the VP4 spike at 4.9 Å resolution (Figure S2A). Unlike previous reconstructions of trypsin digested purified particles, the *in situ* VP4 complex contained additional density adjacent to the prone trunk domain, which could be accounted for by rigid body docking of the head domain of VP4 (Figure 3B, middle).

To determine whether the absence of trypsin during the propagation of rotavirus is sufficient to retain the ordered prone head domain, a separate single particle (i.e., not tomographic) analysis was performed on gradient purified TLPs propagated at MOI = 5 without trypsin (Figures S2B and S2C). Initial icosahedral pose parameters determined for individual viral particles (n = 36,363) were used to extract spike-containing vertices (n = 2,181,780). Multiple rounds of 3D classification and selection were performed until a single class exhibiting a strong density for the VP4 spike was obtained. This class represented only 6% of the total number of vertices, far less than the 90% occupancy seen for intracellular particles, consistent with the known dissociation of the entire VP4 spike during gradient purification.^23^ Further rounds of focused unaligned classification were performed to enrich for particles with the additional density adjacent to the trunk domain of VP4 resulting in a final class consisting of 30,964 sub-particles which represents 25% of all spike-containing sub-particles. Further 3D refinement followed by masking and sharpening yielded a map at 2.7 Å (Figures 3B right and S2A), which enabled an unambiguous rigid body fitting and refinement of the head domain into the density in the prone chain (Figure S2D). This density was higher resolution, but otherwise indistinguishable from that seen on the *in situ* TLPs.

Compared with the upright conformation, the prone head domain is rotated by 117° around y axis slightly offset from the trunk and displaced by 37 Å (Figure 3C; Video S1). Notably, the map enabled modeling of C-terminal residues 222–245 within the trypsin cleavage region of VP4-C head domain. The loop is stabilized by a beta hairpin from the VP4-C trunk domain (residues 414–420), which is folded back compared with its orientation in the two upright monomers (Figure 3C, inset). In this alternate conformation, the two domains share an interaction surface of 844 Å^2^, stabilized via charge complementation (Figure 3D) as well as hydrophobic interactions centered around residues 298, 318, and 320 on the trunk domain and residues 227 and 234 on the head domain. The interacting residues and their positions on the two domains are presented in Figure S2E.

Taken together, these results reveal the configuration of the VP4-C head domain prior to trypsin cleavage and show that its interaction with the corresponding trunk domain occurs through an alternative set of contacts to those occurring in the A and B chains, displacing the domain to one side, away from the tips of the VP4-C trunk domain.

### The enveloped DLP is tethered to the membrane via a trimeric conformation of VP4

The transiently enveloped eDLP is poorly characterized in both its composition and the molecular interactions of VP4 and NSP4. The interaction of the DLP with the ER membrane as it buds into the ER lumen and the acquisition of the VP4 spike are believed to occur simultaneously, creating the transiently enveloped eDLP.^11^ However, the only direct observations available are at low resolution from thin resin-embedded sections of virus infected cells.^24,25^

**Figure 4:**
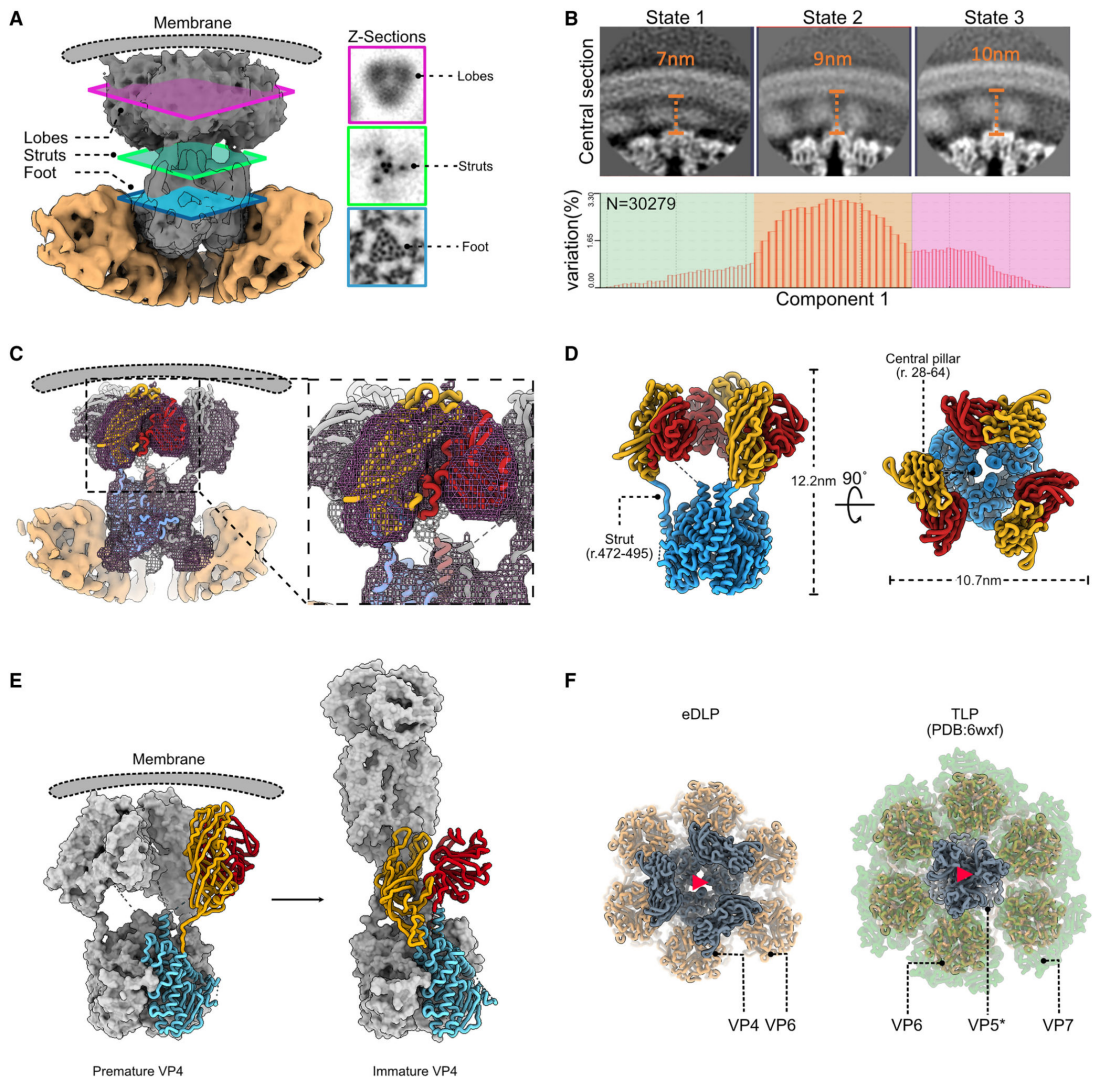
Characterization of VP4 in the eDLP assembly intermediate (A) Overview of the eDLP VP4 density. The VP6 layer is colored in orange with two of the subunits hidden to expose the foot domains of VP4. The membrane layer is represented as a cartoon. Insets show density in the lobes, struts, and foot regions. (B) Flexibility analysis of the pre-mature spike is depicted as central sections through each of the densities. The number of particles in each class is highlighted in the histogram. (C) Map of VP4 after focused classification following multibody analysis. Fit of one of the chains of the pre-mature VP4, colored as in Figure 3A. The membrane and two VP6 subunits have been masked out to aid visualization. The approximate location of the membrane is depicted as a cartoon (gray). (D) Liquorice diagram model of the pre-mature VP4 trimer. (E) Comparison of the pre-mature (left) and extended (right) VP4 molecules. VP4-A and B chains are rendered as surfaces, with VP4-C represented as a liquorice cartoon. (F) Comparison of VP4 from the entry intermediate form of the TLP (PDB: 6wxf, right) with the pre-mature VP4 seen on the eDLP (left). The different protein layers are represented as liquorice cartoons. Red triangle indicates the 3-fold axis.

We observed trimeric densities in the icosahedral map of the eDLP obtained from the wild-type dataset (Figure 2B) that bridge the DLP to the membrane at the approximate positions occupied by VP4. However, the number of subtomograms was too low to obtain a high-resolution map. To better characterize the density at these vertices, the conversion of eDLPs to TLPs was arrested using thapsigargin, an inhibitor of Ca^2+^ concentration regulation in the ER.^26^ Following thapsigargin treatment, 94% of the particles detected (n = 3,124) from 112 tomograms were eDLPs and no TLPs were observed (Figure S3A). Following initial icosahedral orientation estimation, symmetry expansion and co-ordinate re-centering was performed on tri-lobed densities extending from the surface of the capsid. Asymmetric 3D classification followed by further 3D local refinement yielded a map at a nominal resolution of 8.5Å from a final set of 30,278 particles (Figure S3B). Initial rigid body docking of the upright VP4 spike in the density revealed a trimeric foot anchored between VP6 trimers, identical to that found in the TLP (Figure 4A); however, beyond this, the structure is different (Figure S3C), with poorly ordered density forming trimeric lobes of density supported by thin struts of density that extend from the corners of a triangle described by the foot domain (Figure 4A). Additional support is provided by a central “pillar” arising from an extension of the N-terminal helix corresponding to residues 28–64, which was insufficiently ordered to be reliably modeled (Figure 4A). We have termed this intermediate the “pre-mature” VP4 conformation.

Multiple attempts at clarifying the density within the lobes of the pre-mature VP4 trimers using focused refinements did not improve the resolution, suggesting considerable flexibility. Relion multibody analysis did not reveal any specific movements as major contributors to the observed flexibility, rather it appears that a range of motions combine to blur the structure (Figure S3D; Videos S2–S4). The first component describes a radial displacement of the membrane relative to the VP6 layer (Video S2). Projection of the particles along this component enabled partitioning the data into three subpopulations namely state 1, state 2, and state 3 containing 13.7%, 45.7%, and 33.8% of the particles respectively. Our analysis of the radial displacement reveals that the distance between the capsid and the membrane varied by ~3 nm (Figure 4B; Video S2). Further unaligned focused classification of the particles belonging to state 2, used a soft mask that enclosed the three lobes of density revealed one class (15.5% of the particles, Figure S3E) for which the density could be interpreted by rigid body docking of the head and trunk domains of VP4-C derived from TLPs not exposed to trypsin (Figure 4C). The map also enabled modeling of the struts that support the head and trunk domains of VP4 (Figure 4D). Overall, the molecule is 12.2 nm long and 10.7 nm wide (Figure 4D). Compared with its prone form in the TLP, the fitting required an outward tilt of 87° around an axis perpendicular to the surface of the particle (Figure 4E; Video S5). This structure shows some similarities with a trypsin cleaved intermediate that functions during entry^7^ ; however, there are significant differences: first, the outer VP7 layer that caps the intermediate VP6 layer is absent; second, the pre-mature VP4 accommodates the head, trunk, and foot domains, whereas in the entry intermediate only the trunk and foot domains are represented; third, the VP4 domains external to the capsid are more widely spaced in the pre-mature VP4 form (Figure 4F).

In summary, our results have identified a previously unknown conformation of VP4, distinct from those described in either the assembled TLP or during the entry process. This structure is consistent with the existing assembly paradigm in which the viral outer coat proteins are assembled beginning with embedding of VP4 in the VP6 layer when the DLP is budding through the ER membrane and completed by the assembly of the VP7 layer when the transient membrane is lost.^11^

### A single-layered particle lacking viral genome is assembled in the viroplasm

**Figure 5:**
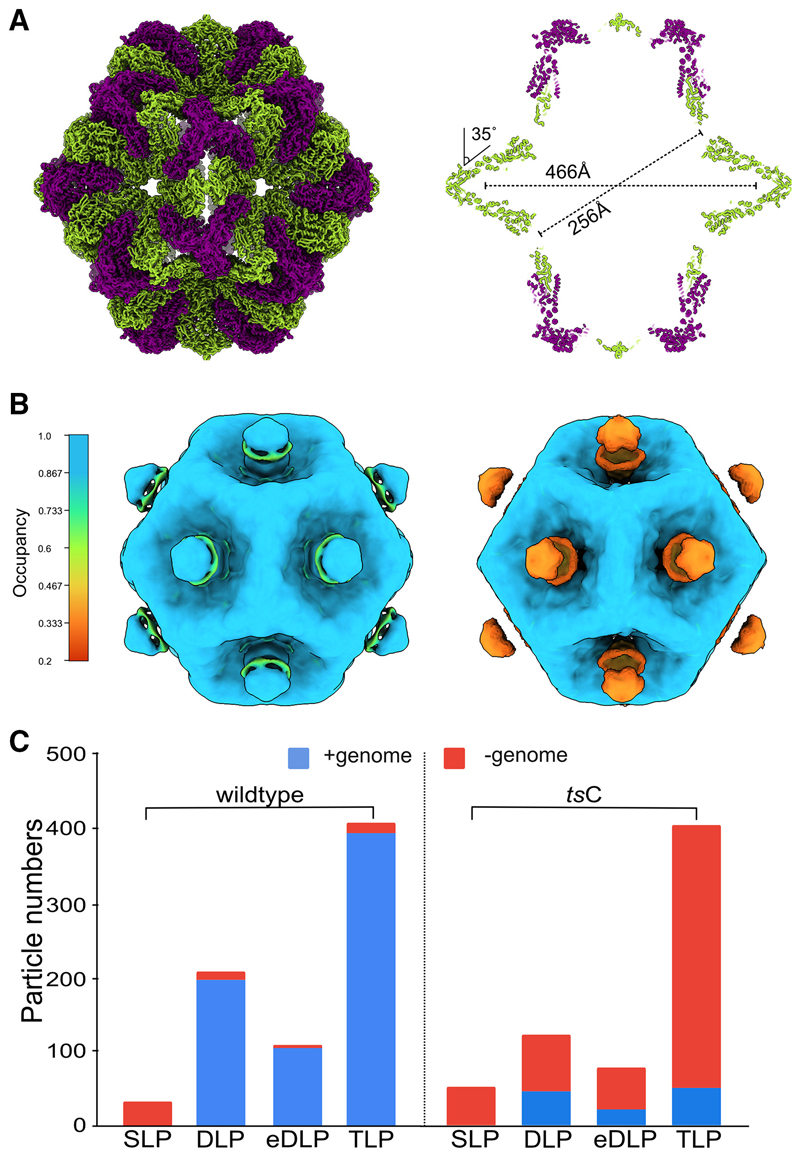
Indented single-layered particle (A) Model of the indented SLP is presented with the outer surface view (left) and a central section (right). The two chains of VP2 A and B are colored in green and purple, respectively. Long and short dimensions of the particle are indicated. The difference in the angle made by adjacent VP2 A in the indented form compared with their position in the expanded form adopted at later assembly stages, is indicated. (B) Isosurface representation of the SLP map from wild type (left) and tsC mutant (right) with the voxels colored by their occupancy. Occupancy of the blobs in the SLP maps was estimated in a resolution-independent manner using the relative contrast of the voxels within the map.^27^ (C) Comparison of the number of genome-containing and genome-less particles in the wild-type virus versus the conditionally lethal tsC mutant.

While the DLP and TLP assembly stages of rotavirus have been well studied, earlier assembly stages have not been detected.^24^ Our previous study of the assembly of the turreted mammalian orthoreovirus within infected cells revealed the presence of an abundant SLP within virus factories that was indented at the 5-fold symmetry axes and lacked the genome.^16^ We observe an equivalent genome-less SLP in rotavirus viroplasms (Figures 1D, 1I, and 2D). Of 85 tomograms, 6 captured distinct regions of viroplasm that contain the early assembly stages up to the formation of the DLP. Within the viroplasms 32 rotavirus SLPs were found, none of which contained sufficient density to account for the presence of either the single-stranded or double-stranded form of the genome (Figures 1D, 1I, and 2D). In contrast 207 DLPs/partially eDLPs were found, 95% of which had packaged the genome. Using STA, a map at 27-Å resolution was obtained from 28 SLPs permitting unambiguous rigid body docking of the VP2A-B dimer into the density (Figure 2E). The gross structure of the VP2 shell of the rotavirus SLP is similar to the equivalent lambda 2 shell of the reovirus SLP (Figure 5A).^16^ As found with reovirus the VP2A-B dimers at the 5-fold were indented by approximately 35° relative to their orientation in the DLP, eDLP, and TLP. The resulting structure has a minimum diameter of ~256 Å, maximum diameter of ~466 Å, and internal volume of ~10^7^ Å^3^ (Figure 5A).

The only significant densities in the SLPs that were not explained by VP2 were substantial blobs of density on the outside of the indented 5-fold vertices (Figure 5B). The volume of these blobs was consistent with them corresponding to single copies of the VP1 polymerase, and intriguingly, the polymerase was also found at the 5-fold axes in the next observed assembly stage, the DLP, but here, it was found inside the VP2 layer, suggesting a potential role for VP1 at this stage of assembly (Figure S4A). To investigate directly whether VP1 participates in the assembly of the SLP, we used the tsC-mutant isolate containing a temperature-sensitive variant of the VP1 protein at the non-permissive temperature of 39°C.^28^ The formation of empty SLPs was recapitulated, with all 52 examples of SLPs lacking the genome, but in contrast to wild-type rotavirus, the DLP, eDLP, and TLP assembly stages of tsC were also found to lack the genome in either the single-stranded or double-stranded form in most particles (Figures 5C, S4B, and S4C–S4F). Furthermore, the densities on the outside the 5-fold vertices were reduced in intensity in the *ts*C mutant to ~30% of the wild type (Figure 5B). This progression of genome-less capsids through the later stages of assembly shows that the recruitment of the 11 single-stranded genome segments is not essential to trigger the next stage in the assembly pathway.

## Discussion

**Figure 6:**
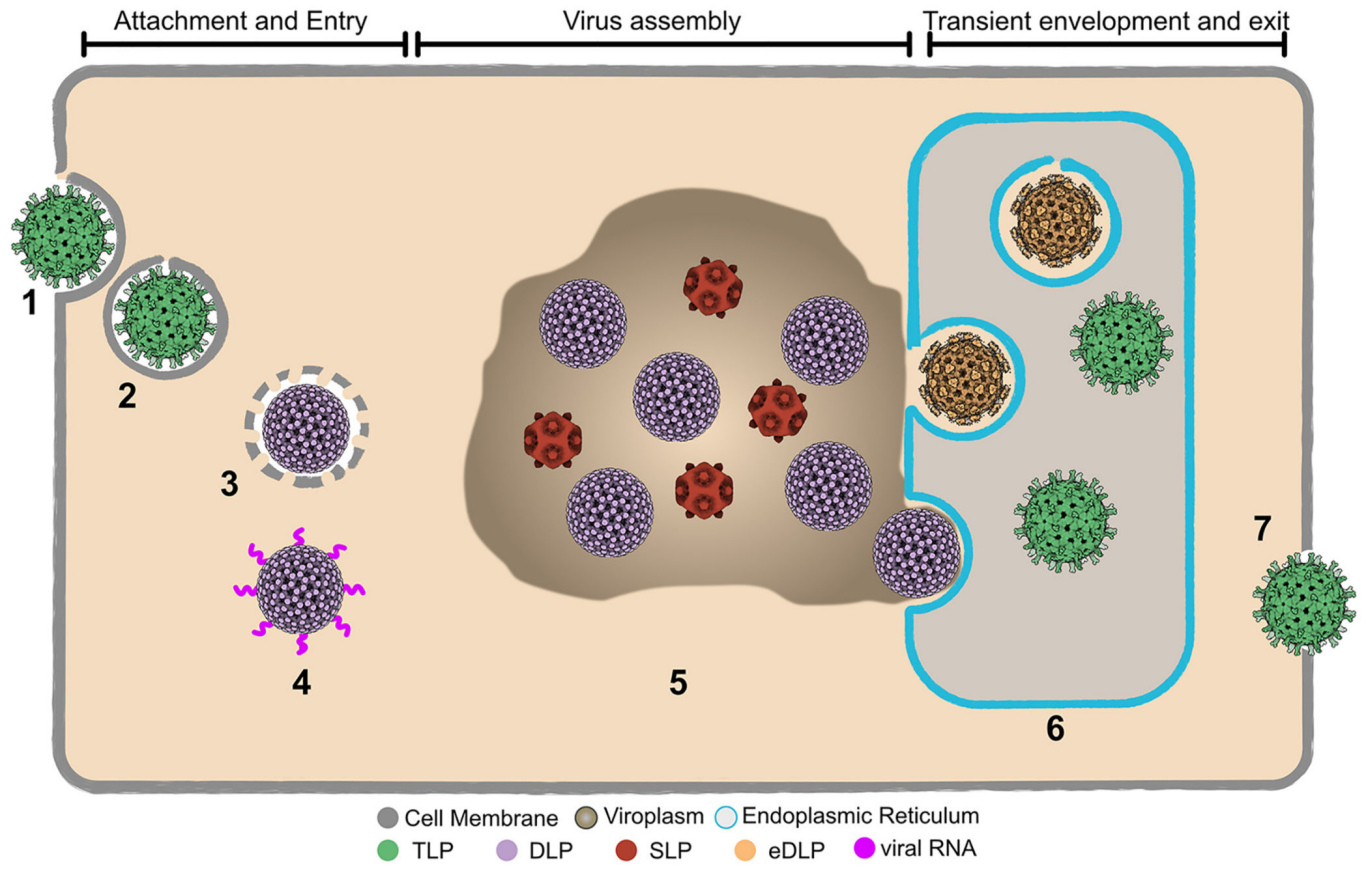
Assembly intermediates in the rotavirus replication cycle Infection occurs following virus entry and the release of the incoming DLP into the cytoplasm where primary transcription occurs (1–4). Following viroplasm formation and synthesis of viral structural proteins, virus assembly begins with the indented SLPs which are converted to the DLP stage (5). The DLP acquires VP4 as it buds through the ER membrane gaining a transient envelope. Loss of the envelope and the rapid acquisition of the VP7 layer leads to the formation of the immature TLP in the ER lumen (6). The fully assembled virions are released into the extracellular medium where exposure to trypsin in the small intestine converts them to mature infectious virions (7).

The cryo-ET analysis of rotavirus-infected cells has enabled the direct visualization of events within the viroplasm and ER at molecular resolution without sample preparation artefacts. Specifically, subtomogram refinement and averaging has allowed the *in situ* characterization of discrete stages of rotavirus assembly, including stages that cannot be purified due to their inherent low stability, as shown in Figure 6, which identifies the four particles we have analyzed in detail.

The early stages of viral assembly, up to the DLP, occur in the viroplasm. Here, we show that *in situ* DLPs are located exclusively in the viroplasm and are structurally indistinguishable from those purified from infected cells. However, we also identified in the viroplasms an earlier assembly intermediate not previously observed, the indented SLP, which due to its thin wall and the absence of the genome is much harder to detect and likely unstable. These SLPs are remarkably similar to the SLP intermediates previously described inside reovirus virus factories and are reminiscent of an early assembly stage characterized in the bacteriophages phi6/8 of the *Cystoviridae* family.^29,30^ The A and B VP2 subunits, which form the icosahedral SLP^16^ necessarily interact using different contacts in the indented SLP compared with the expanded arrangement seen in the later assembly stages. Due to their low abundance, the resolution of the rotavirus SLPs is lower than that obtained for reovirus SLPs. However, the fold of the inner shell protein is well conserved across the *Reoviridae* family and the alpha helices that participate in the two alternative sets of contacts in reovirus lambda 2 are also present in the equivalent positions in rotavirus VP2, indicating that the two states of expansion are likely achieved by a similar helix rachet mechanism.^16^ These highly similar geometries and arrangements found at the equivalent stage of assembly indicate that the indented pre-packaging state is a conserved assembly intermediate found across the *Reoviridae* and *Cystoviridae* families. This suggests that high-level structure-based taxonomy, already applied to some DNA viruses, may also be appropriate to RNA viruses.^31^

Surprisingly, the rotavirus SLP has pronounced blobs of density at the 5-fold axes on the outer surface of the indented VP2 shell that are consistent with the size of the polymerase (Figure 5B). For the tsC mutant a change (L138P) in the polymerase leads to its mis-localization from the viroplasm to the cytoplasm at non-permissive temperatures and reduces the occupancy of the blobs by a factor of three (Figures 5B and S4A). The reduction of density at these sites is accompanied by a high failure rate in packaging/replicating the genome as seen in the absence of RNA in the assembling particles (Figures 5C and S4B). The large increase in genome-less particles observed at later assembly stages (DLP, eDLP, and TLP), without a corresponding reduction in the number of particles, shows that when packaging fails through the defect in VP1, progression to the later stages is not delayed indefinitely (Figures S4B–S4F). Indeed genome-less DLPs, eDLPs, and TLPs were observed, albeit at a much lower frequency, in the wild-type SA11 strain, indicating a failure of the natural packaging in ~5% of particles (Figure 5C). Furthermore, in the minority of cases where genome was present in the later assembly stages of the tsC mutant at 39°C, the density observed was always consistent with dsRNA rather than ssRNA, showing that the *ts* lesion in VP1 affects recruitment of the genome but does not prevent the synthesis of the negative strands. Together, these findings add to the biochemical characterization of the replicase function of VP1 when bound to VP2 and support the proposal that its specific binding of the 3′ end of the viral transcripts allows it to participate in the recruitment of the eleven genome segments at a stage prior to synthesis of the negative strands.^32,33^ The loss of the polymerase on the SLP prior to negative strand synthesis offers a molecular understanding for the reduction in dsRNA synthesis previously reported for the tsC mutant.^28,34^ The location of the polymerase on the outside of the genome-less SLP raises further questions regarding how the polymerase-transcript complexes are translocated into the assembling particle. The flexibility of the VP2 shell that allows it to transition between an indented an expanded form suggest that they may move directly through the gap at each 5-fold between the five VP2 A subunits. While the structural data do not speak to the question of how a single copy of each genome segment is selected, we propose that following VP1 binding of viral transcripts at their 3′ end,^32,33^ the VP1-transcript complexes associate with the outer face of the SLP at the indented 5-fold, prior to their assortment, packaging and replication through processes that are yet to be uncovered. The *in situ* visualization of the genome-less SLP within viroplasms, in combination with the lack of intermediate forms between the SLP and DLP suggest the pre-packaging SLP stage represents a pause in the assembly pathway followed by a rapid conversion to the dsRNA-containing DLP stage after genome packaging.

The next stage in assembly occurs when DLPs leave the viroplasm and bud into the adjacent ER, in the process gaining VP4 and a transient envelope, to form eDLPs. We found that the DLP is tethered to the envelope by VP4 (Figure 4A) and that VP4 adopts a flexible but broadly 3-fold symmetric pre-mature structure (Figures 4B–4D). This undescribed conformation shares some features with an intermediate structure captured during entry in that the foot domain of VP4 is present in both conformations (Figures 4A and S3C).^7^ However, in the eDLP the capsid distal domains reach the membrane and are linked flexibly to the foot via residues 491–498 (Figures 4A and 4C). The transition from this form to an intracellular TLP involves gross changes in the conformation and arrangement of the three VP4 subunits as well as the addition of the VP7 layer and loss of the transient envelope. The extension of VP4 into the upright elongated “dimeric” conformation, stabilized by increased lateral interactions between the two elongated subunits, would likely breach the membrane. VP7 is proposed to be associated with this membrane and so this could allow VP7 trimers to assemble and form the outer layer of the TLP. The absence of any examples of the eDLP captured part way through this transition suggests that the process of losing the membrane and acquiring the VP7 layer occurs rapidly. This is consistent with the model of assembly locating VP7 on the outer surface of the eDLP envelope, available to form the outer capsid layer simultaneous with the loss of the envelope. Although neither VP7 nor NSP4 can be seen directly in the eDLP map, they are probably simply poorly ordered with respect to the rigid VP6 layer. In addition, the changes in the local environment of VP4 caused by the acquisition of the VP7 layer may stabilize the VP4 trimer in its final asymmetric conformation. The addition of VP7 may therefore catalyze the conversion of VP4 molecules to the mature “dimeric spike” state. However, we note that our work raises further questions, for instance, what is the trigger for the initial membrane-breaching VP4 conversion and is this event facilitated by interactions of pre-mature VP4 at the membrane?

**Figure 7:**
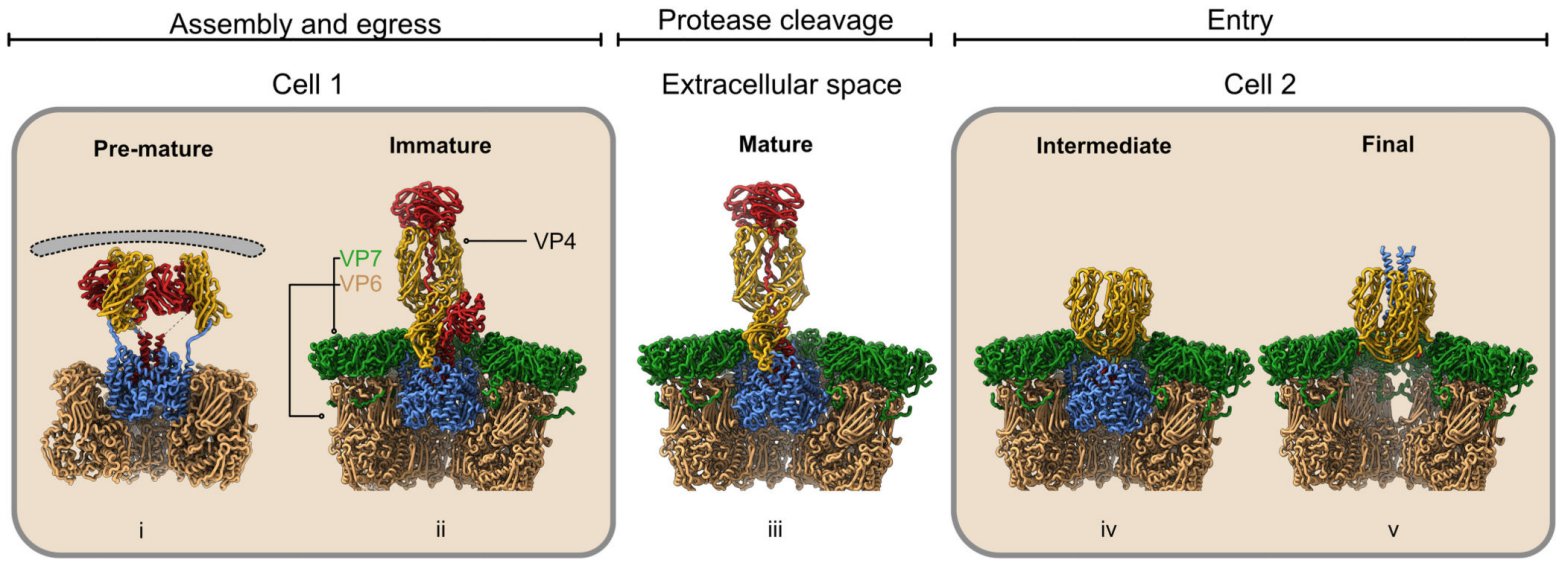
Conformations of VP4 sampled during entry and maturation stages Schematic of the transitions occurring in VP4 during maturation, egress, and entry beginning with (i) the insertion of VP4 into the VP6 layer in the transiently enveloped stage. (ii) The upright dimeric VP4 conformation occurs after the immature TLP is released into the ER lumen. (iii) Extracellular proteases present in the gut lumen cleave VP4 and render the TLP competent for entry. (iv and v) Following attachment VP4 undergoes gross conformational changes via an intermediate conformation in which the foot domain is present and culminating with the final entry-competent conformation in which the foot domain is disordered.^7^

From our work and previous studies on rotavirus cell entry, we can now see that VP4 undergoes two sets of large-scale conformational changes, first, during the loss of the transient viral envelope during morphogenesis and second, on entry to the host cell, initiating the next infectious cycle. These achieve very different outcomes. In the first an unknown trigger, possibly associated with the concentration of VP7 on the envelope, causes the reasonably stable, membrane-interacting, pre-mature form of uncleaved VP4 in the eDLP to undergo concerted conversion from flexible trimeric structures to asymmetric “dimeric” spikes around which the VP7 layer is laid while the membrane is rapidly lost. In contrast, during cell entry, these VP4 spikes interact with the cell membrane, triggering cell entry and the loss of both VP4 and VP7. Reversion to the pre-mature VP4 structure at this stage is impossible since trypsin cleavage has led to the loss of a head domain from one of the subunits (Figure 7). Further understanding of how common structural elements of VP4 switch between symmetric and asymmetric structures to drive different conformational changes and control key aspects of the virus assembly and life cycle requires a reductionist approach to clarify the stability and dynamics of the various protein and membrane structures in the different biochemical environments in which they occur.

Lastly, we find that the structure of the non-infectious intracellular rotavirus TLP differs from the fully infectious mature virion. The activation of the virion is caused by trypsin digestion in the small intestine of sites in the loop connecting the head and trunk domains of VP4 (Figure 7). However, it is not well understood how the cleavages within VP4 result in greater infectivity. We found in the intracellular TLP additional density associated with VP4, likely corresponding to the missing part of one subunit in the cleaved TLP, as suggested by a previous report of purified TLPs prepared without trypsin.^22^ We therefore determined a high-resolution structure of purified, uncleaved particles to test this interpretation. The structures derived from *in situ* TLPs and gradient purified TLPs without exposure to trypsin unambiguously show the C-subunit head domain located at the base of the uncleaved VP4 spike and that this form of VP4 exists within the ER in nascent TLPs (Figure 3B). The high-resolution map obtained from purified TLPs allows the positioning of the head domain with sufficient precision relative to the trunk domain to characterize the charge-based and hydrophobic interactions stabilizing the arrangement of these domains via an alternative and significantly smaller interaction surface than in the upright conformation (Figure 3D). The population of purified particles bearing the extra head domain represented a minority of particles and the local resolution of this region was less than the rest of the capsid, most likely due to flexibility resulting from the small interaction surface. We found a significantly higher VP4 occupancy in TLPs within the ER compared with gradient purified TLPs, consistent with the finding that the VP4 spikes tend to be lost during routine CsCl-based purification, with the strain of rotavirus, the use of organic solvent during the purification, and its proteolytic cleavage state all reported to influence the proportion of VP4 retained.^23^

In summary, by integrating genetic and inhibitor-based methods to modulate rotavirus assembly with the capture of intermediates *in situ* using cryo-ET and STA, we have detected and characterized at the molecular level assembly stages not previously detectable. The direct observation of these events inside the infected cell with undisturbed cellular environment provided the context at the nanometer scale of how the assembly events relate spatially to the cellular structures in which they occur and allowed the study of delicate, short-lived forms that cannot be purified outside of their native milieu. We believe this integrated approach has great potential to understand in atomic detail and at the level of cellular ultrastructure a wide range of both viral and non-viral cellular processes occurring inside living cells.

## Star★Methods

Detailed methods are provided in the online version of this paper and include the following:

- KEY RESOURCES TABLE
- RESOURCE AVAILABILITY
  ○ Lead contact
  ○ Materials availability
  ○ Data and code availability
- EXPERIMENTAL MODEL AND SUBJECT DETAILS
  ○ Cell lines
  ○ Rotavirus isolates
- METHOD DETAILS
  ○ Purification of TLPs produced without trypsin
  ○ SDS PAGE analysis
  ○ Cell preparation for FIB-milling and cryo-ET
  ○ Focussed ion beam milling
  ○ Cryo-ET data acquisition
  ○ Tomogram reconstruction and subtomogram averaging of whole particles
  ○ Flexibility analysis of VP4
  ○ Sample preparation and data collection of purified TLP
  ○ Cryo-EM processing of purified TLP
  ○ Model building, analysis and validation

## Star★Methods

### Key Resources Table

**Table:** 

REAGENT or RESOURCE	SOURCE	IDENTIFIER
Bacterial and virus strains		
Rotavirus SA11	Takeshi Kobayashi	N/A
Rotavirus SA11 tsC	Sarah McDonald	N/A
Chemicals, peptides, and recombinant proteins		
Glasgow’s MEM	Thermo Fisher	Cat# 21710
Fetal Bovine Serum	Thermo Fisher	Cat# A5256701
Trypsin-EDTA	Thermo Fisher	Cat# 25300096
L-glutamine	Thermo Fisher	Cat# 25030024
HEPES, Free Acid	Sigma Aldrich	Cat# H3375
CaCl2	Sigma Aldrich	Cat# C5670
NaCl	Sigma Aldrich	Cat# S9888
Tris base	Sigma Aldrich	Cat# T1503
Vertrel-XF	Sigma Aldrich	Cat# 94884
2 Mercaptoethanol	Sigma Aldrich	Cat# M6250
CsCl	Thermoscientific	Cat# J65950.XK
Thapsigargin	Thermo Fisher	Cat# T7458
Deposited data		
The coordinates and maps in this paper are available via the PDB and EMDB databases respectively.	This study	PDB:8BP8 (in vitro TLP spike), PDB:8COA (in situ TLP spike), PDB:8CO6 (in situ TLP penton). EMDB accession codes: EMDB-16146 (in vitro TLP spike), EMDB-16774 (in situ TLP spike), EMDB-16773 (in situ TLP (icosahedral)), EMDB-16772 (in situ TLP (penton)), EMDB-16771 (eDLP (icosahedaral)), EMDB-16767 (eDLP (penton)), EMDB-16768 (DLP (icosahedral)), EMDB-16769 (DLP (penton)), EMDB-16772 (SLP (icoshaderal))
Experimental models: Cell lines		
MA104 cells	Malcolm McCrae	N/A
Software and algorithms		
PHENIX	Liebschner et al., 2019^35^	https://www.phenix-online.org/
COOT	Emsley and Cowtan^36^	https://www2.mrc-lmb.cam.ac.uk/personal/pemsley/coot/
ChimeraX	Pettersen et al.^37^	https://www.cgl.ucsf.edu/chimerax/
Relion 3.1.3	Zivanov et al.^38^	https://github.com/3dem/relion/releases
Warp/M 1.0.9	Tegunov et al.^17^	http://www.warpem.com/warp/
IMOD	Mastronarde and Held^39^	https://bio3d.colorado.edu/imod/
Tomo 5	ThermoFisher	N/A
Other		
This work was performed with the support of Diamond Light Source and the electron Bio-Imaging Centre (eBIC), instrument eBIC Titan Krios III (proposal nt21004-432, nt21004-488, nt21004-611, NT21004-290, EM20223-27) and instrument Titan Krios II (proposal EM20223-60).	This paper	https://www.diamond.ac.uk/covid-19/for-scientists/rapid-access.html

### Resource Availability

#### Lead contact

Further information and requests for resources and reagents should be directed to and will be fulfilled by the lead contact, David Stuart, david.stuart@strubi.ox.ac.uk

#### Materials availability

This study did not generate new unique reagents.

### Experimental Model and Subject Details

#### Cell lines

MA104 cells – Immortal kidney epithelium cell line derived from African green monkey. Maintained in Glasgow minimal essential medium (GMEM) supplemented with 5% (v/v) foetal bovine serum at 37°C in 5% CO2. Not authenticated.

#### Rotavirus isolates

SA11^40^ – Routinely propagated in MA104 cells in Glasgow minimal essential medium (GMEM) supplemented with trypsin (2μg/mL) at 37°C in 5% CO2. To produce virus in the absence of trypsin SA11 was propagated without added trypsin and with 5% (v/v) foetal bovine serum at 37°C in 5% CO2.

SA11 tsC^28^ (kind gift from Sarah McDonald) – Routinely propagated in MA104 cells in Glasgow minimal essential medium (GMEM) supplemented with trypsin (2μg/mL) at 31 °C in 5% CO2. To display the temperature-sensitive phenotype an incubation temperature of 39°C was used.

### Method Details

#### Purification of TLPs produced without trypsin

MA104 cells were infected at a multiplicity of infection (MOI) of 5 in GMEM supplemented with 5% (v/v) foetal bovine serum in the absence of trypsin at 37°C for 48 hours. The cells and growth medium were centrifuged in 2L pots at 6,000g 4°C for 20 minutes to pellet cells and cell debris. The pellet was extracted with Vertrel-XF (DuPont) to extract the lipid fraction and centrifuged at 4,000g 4°C for 5 minutes. The aqueous phase containing the TLPs was centrifuged at 10,000g 4°C for 10 minutes to pellet residual cell debris. The supernatant was centrifuged at 62,000g (r_ave_) 4°C for 1 hour to pellet the TLPs, and resuspended in 50mM Tris HCl pH8.0, 50mM NaCl, 1.5mM beta-mercaptoethanol, 5mM CaCl_2_. The TLPs were separated from DLPs and other material by CsCl gradient centrifugation in the presence of 5mM CaCl_2_ as described previously.^41^ TLPs were collected by side puncture and concentrated by ultracentrifugation at 110,000g (r_ave_) for 1 hour at 12°C and resuspended in 20mM Tris HCl pH8.0, 140mM NaCl, 2mM CaCl_2_. Stored at 4°C.

#### SDS PAGE analysis

Samples were separated on 4-12% NuPAGE Bis-Tris gels (Invitrogen) in MES running buffer (Invitrogen).

#### Cell preparation for FIB-milling and cryo-ET

Glow-discharged carbon-coated gold TEM grids (Quantifoil R2/1) were seeded with 33,000 MA104 cells and allowed to adhere for 4 hours at 37°C. Cells were infected at an MOI of 10 and incubated at 37°C for wildtype virus, or at the non-permissive temperature, 39°C, for the temperature-sensitive VP1 mutant (tsC). Treatment of wildtype virus with thapsigargin when required was done at 2 hours post-infection at 750nM final concentration. At 13 hours post-infection (hpi) the grids were flash-frozen in liquid ethane by blotting from the back side for 6s using a Leica GP2 plunger (Leica Microsystems).

#### Focussed ion beam milling

FIB milling was performed similar to previously established methods.^16^ Briefly, plunge-frozen grids containing infected cells were clipped into auto-grid rims (ThermoFisher Scientific) and loaded into a Scios DualBeam system (ThermoFisher Scientific) using a Quorum cryo transfer station (PP3010T) equipped with a cryo stage cooled to –168 °C. Prior to milling, grids were sputter coated with platinum in the PP3010T chamber and then coated with a layer of organoplatinum (trimethyl(methylcyclopentadienyl)platinum(IV)) using the gas injection system (incorporated in the Scios chamber). Milling was performed in a stepwise fashion, using a 30 kV Ga^+^ beam, and currents of 300 to 50pA. Final thinning was performed at beam currents between 45 and 30 pA to generate a lamella with a thickness of less than 200 nm. Progress of the final milling was monitored with the scanning electron microscope operated at 2 kV and 6.3 pA.

#### Cryo-ET data acquisition

Tilt series were collected on several positions from three different lamellae using a Thermo Scientific Krios electron microscope (eBIC Krios III) operating at 300 keV and equipped with a SelectrisX (Thermo Scientific) post-column energy filter (selecting a 5eV window) on a Falcon 4 direct electron detector (Thermo Scientific). Data acquisition was performed covering an angular range from –40° to +40° with 2° angular increments recorded automatically using the dose-symmetric tilting scheme^42,43^ under low-dose conditions using Tomo 5 (Thermo Scientific). Each tilt series was collected with a nominal defocus value between 1.5 and 3.5μm. Each tilt point was acquired as movies (containing 8 frames) in counting mode using a dose of 2.2 e–/Å^2^ per tilt. The total cumulative dose for each tilt series was 90 e–/Å^2^. Magnification was nominally 64,000x, with a calibrated pixel size of 1.97 Å/px.

#### Tomogram reconstruction and subtomogram averaging of whole particles

In total 85 tilt series were recorded from the wild-type infected cells, 127 from the drug-treated and virus infected cells and 53 from cells infected with *ts*C mutant (Table S1 and S2). Warp (v1.0.9)^17^ was used to perform reference-free movie-frame alignment, CTF estimation and defocus estimation on individual tilt frames as well as generation of dose-weighted tilt-series stacks. The dose-weighted stack was then imported into IMOD (4.11)^39^ and tilt-series alignment was carried out using patch-tracking. At this stage tilt series with poor alignment statistics (wt:28, drug-treated:12) were excluded from further processing. The alignment parameters were imported into Warp and CTF estimation was carried out by fitting a tilt-angle constrained per-tilt CTF model implemented in the package. Tomogram denoising was performed using the Noise2Map routine implemented within Warp.^17^ Binned, denoised tomograms (10Å/px) were generated in Warp and used to train a convolution neural network for particle picking, implemented within the crYOLO^44^ (v1.8) software package. The resulting coordinates were manually examined in IMOD, and false positives removed. Remaining coordinates were then used to extract sub-tomograms (particles) resampled to 2.4Å/px using Warp. The extracted whole virus particle sub-tomograms were then subjected to a single round of 3D classification in Relion 3.1.2.^38,45^ Each class representing a stable assembly intermediate was individually 3D-refined. The resulting pose estimation of each particle was then imported into M to perform iterative reference-based tilt-series refinement and generate high-resolution maps of each assembly intermediate. Icosahedral symmetry was imposed throughout the process. To permit deviations from the icosahedral geometry, subparticle refinement was performed by focusing on the five-folds of the viral capsids. Briefly, the icosahedral optimised poses for each of the particle types were symmetry expanded using *relion_particle_symmetry_expand* and filtered such that 12 poses were generated for each icosahedral particle, each centring one of the 12 C5-symmetric pentagonal faces of the viral capsid. These were re-extracted via Warp at the full resolution (1.97Å/px) and subjected to a round of 3D classification in Relion without imposing any symmetry. Only classes with presence of strong secondary structure were retained and subjected to 3D-refinement of poses under C5 symmetry. The results from the preceding step were imported in M and the refinement process was repeated as described above. The indented SLP was not analysed in the same way, as too few such particles were identified. A similar approach was used to isolate and refine the VP4 spikes from the eDLP and TLP intermediates.

A summary of cryo-ET data acquisition and data processing is presented in Tables S1 and S2.

#### Flexibility analysis of VP4

We used the multi body refinement approach in RELION 3.1.2 to analyse the variability in relative position of membrane and VP4. First a consensus refinement was focused on the capsid assembly pentagons. Two bodies (the capsid and membrane) were defined in this consensus reconstruction by defining two hemispherical masks with a soft edge of 20 pixels. Multi-body refinement^46^ was performed on a box size of 128 pixels at a pixel size of 2.4Å/px with an angular sampling set to 1.8° and translational sampling of 21 pixels with a step size of 7 pixels.

#### Sample preparation and data collection of purified TLP

Quantifoil R2/1 copper grids were pre-coated with graphene oxide (GO) using a previously established protocol.^47^ Purified TLP’s (3.5μL) at a concentration of 7mg/ml was applied on GO-coated grids before being plunge frozen using a Leica GP2 freezing robot (Leica GmBH). Grids with optimal ice thickness were imaged in a Titan Krios equipped with a Gatan K2 detector and an energy filter. The slit width was set at 20eV. A total dose of 39.8 e^-^/Å^2^ was partitioned across 40 frames. Data was acquired at a calibrated pixel size of 0.82 Å/px and nominal magnification of 165,000x.

#### Cryo-EM processing of purified TLP

Single particle averaging was performed within the Relion 4.0 framework.^48^ Movies were motion corrected using Relion’s implementation with a default B-factor of -150.^49^ Global CTF parameters were determined using CTFFIND (v4.1.14)^50^ on dose-weighted frames. Particle picking was performed using Cryolo (v.1.8.0).^44^ False-positive picks and junk particles were discarded by performing single round 2D classification in Relion on a four times down sampled image stack followed by a single round of 3D classification. This yielded a final stack of 36,363 particles. 3D icosahedral refinement followed by CTF Refinement, higher order aberration correction and Bayesian polishing was then performed on the final clean particles. Symmetry expansion and particle subtraction was then used to isolate the vertices containing the VP4 spikes in a box of 448px. Local refinement was performed on the VP4 spike to allow deviations from the icosahedral symmetry that was imposed in the previous step. This yielded a consensus average with an average global resolution of 2.7Å. Relion was then used to produce a final map that was filtered by local resolution estimates.

#### Model building, analysis and validation

PDB:6WXE^7^ served as an initial template model. First, it was rigid body docked into the asymmetric unit of the *in vitro* purified TLP virion reconstruction. Individual chains were built and real space refined in Coot v.0.9.8^36^ followed by refinement in Phenix v.1.20.^51^ The refinement statistics are presented in Table S3. The refined model was then used to perform rigid body fitting into the assembly intermediates attained by subtomogram averaging. Modelling of the trimeric eDLP VP4 was performed by placing the VP4-C chain by hand in the density and using the fitmap function in ChimeraX^37^ to refine the fit in the density. The loop between trunk and foot domains was built in Coot using its fit loop function. Finally, the model was symmetry expanded to model the remaining two domains of the molecule using the symmetry function in Chimera.^52^

Modeling of the rotavirus VP2 dimers of the SLP was performed by first rigid body fitting the reovirus SLP (PDB:6X7F) asymmetric unit into the density. Next, the rotavirus VP2 asymmetric unit was least squared superposed using the matchmaker function in ChimeraX. Finally, the rotavirus VP2 asymmetric unit was symmetry expanded to include all subunits of the icosahedral shell.

## Supplementary Material

Document S1. Figures S1-S4 & Tables S1-S3

Video S1

Video S2

Video S3

Video S4

Video S5

## Data Availability

Models and maps are deposited in the PDB and EMDB respectively. PDB accession codes are PDB: 8BP8 (in vitro TLP spike), PDB: 8COA (in situ TLP spike), PDB: 8CO6 (in situ TLP penton). EMDB accession codes are: EMDB-16146 (in vitro TLP spike), EMDB-16774 (in situ TLP spike), EMDB-16773 (in situ TLP (icosahedral)), EMDB-16772 (in situ TLP (penton)), EMDB-16771 (eDLP (icosahedaral)), EMDB-16767 (eDLP (penton)), EMDB-16768 (DLP (icosahedral)), EMDB-16769 (DLP (penton)), EMDB-16772 (SLP (icoshaderal)). This paper does not report original code. Any additional information required to re-analyze the data reported in this paper is available from the lead contact upon request.

## In brief

Shah et al. used cryo-tomography to characterize the assembly pathway of rotavirus inside infected cells. The use of flash frozen samples preserved the assembly stages in their native state and relative proportions, and subtomogram averaging revealed their molecular structures, to near atomic detail in the best case.

## Highlights

- Cryoelectron tomography enabled characterization of rotavirus assembly intermediates
- Subtomogram averaging provided detailed descriptions of the intermediates
- Tri-lobed conformation of intact attachment protein VP4 described in enveloped stage
- RNA-dependent RNA polymerase, VP1, has a role in recruiting genomes to particles
