# Supplementary material for: Characterization of the rotavirus assembly pathway in situ using cryoelectron tomography
Source: Cell Host Microbe. Author manuscript; Available in PMC 2023 Dec 5. (PMC7615348; doi:10.1016/j.chom.2023.03.004)
Supplement: Document S1. Figures S1-S4 & Tables S1-S3 [file EMS191518-supplement-Document_S1__Figures_S1_S4___Tables_S1_S3.pdf]

Cell Host & Microbe, Volume *31*

Supplemental information

Characterization of the rotavirus assembly pathway *in situ* using cryoelectron tomography

Pranav N.M. Shah, James B. Gilchrist, Björn O. Forsberg, Alister Burt, Andrew Howe, Shyamal Mosalaganti, William Wan, Julika Radecke, Yuriy Chaban, Geoff Sutton, David I. Stuart, and Mark Boyce

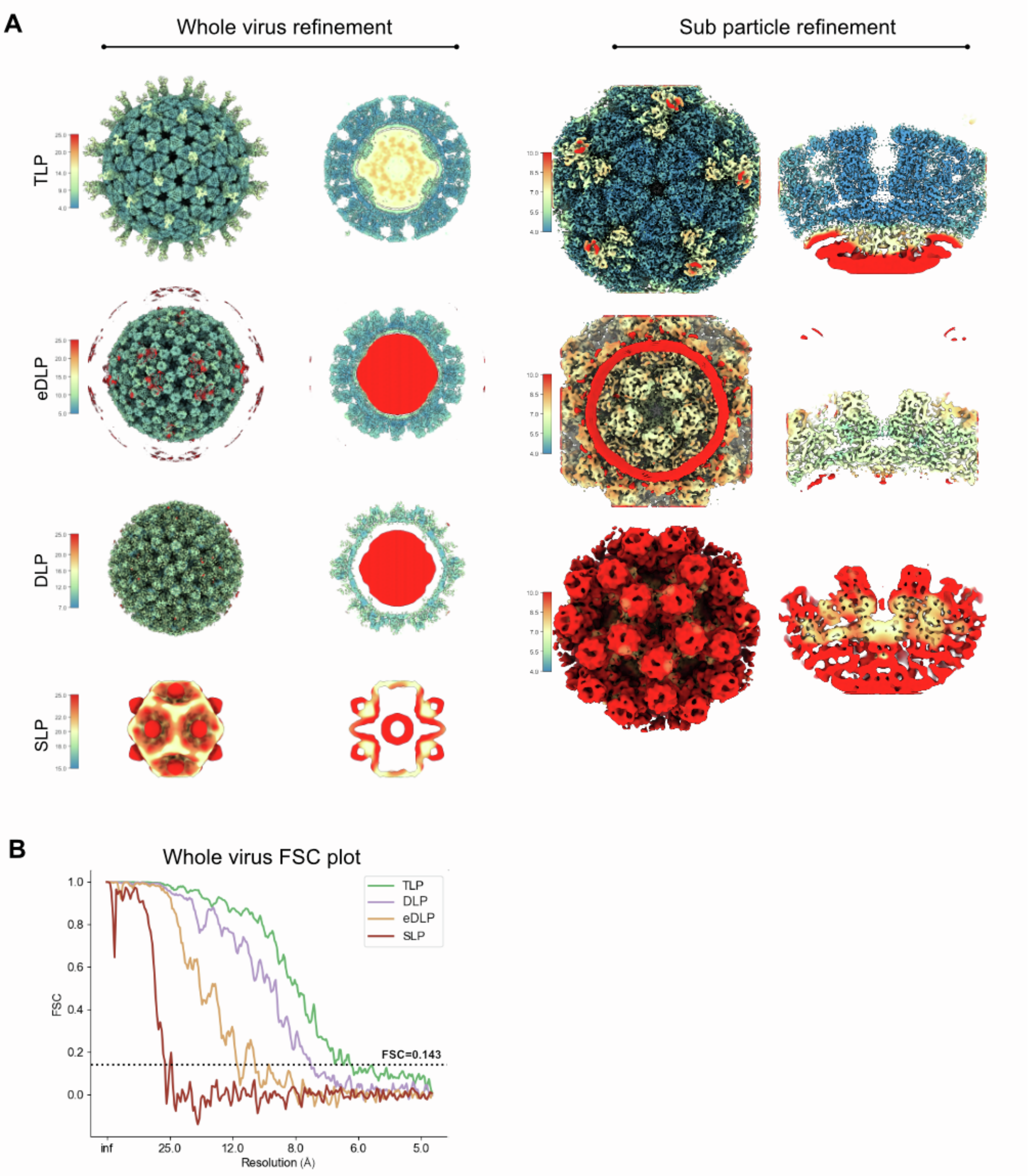

Figure S1. Local resolution estimates of subtomogram averages. (A) Isosurface representations of icosahedral (left) and pentamer (right) densities are coloured by their local resolution. (B) FSC traces for the icosahedrally averaged densities. Related to Figure 2

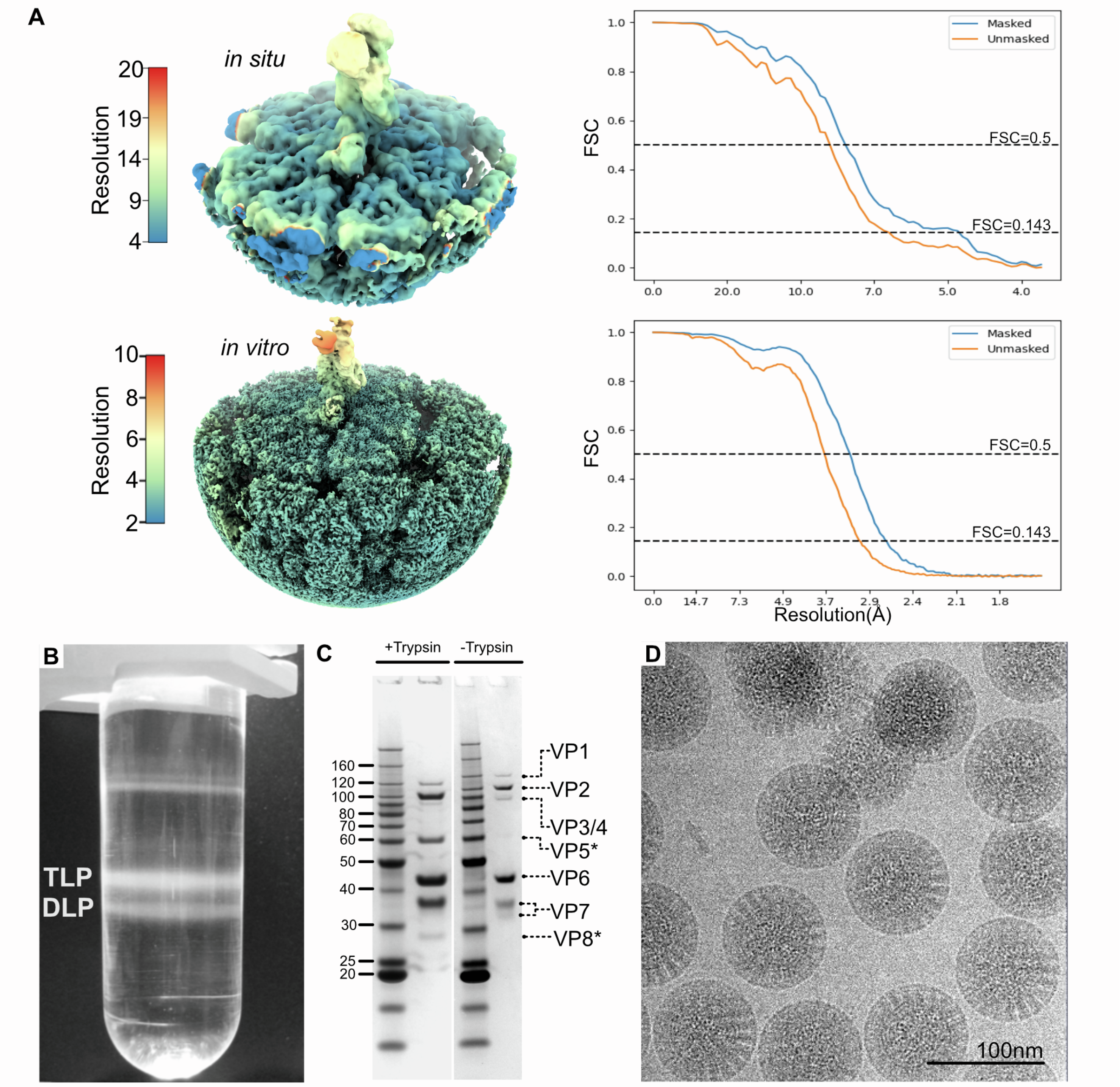

E

Resid ID	Trunk	Resid ID	Head
282	VAL	70	GLN
283	LYS	87	THR
284	SER	88	ALA
285	GLY	89	ALA
286	GLY	109	GLU
292	SER	111	ASN
293	GLU	112	VAL
296	PHE	117	ARG
298	PRO	135	GLN
299	ALA	138	TRP
300	ASN	163	LYS
317	THR	226	PRO
319	SER	227	ILE
320	VAL	228	GLN
321	ASN	231	ARG
323	MET	232	ASN
332	SER	233	ILE
352	TYR	234	VAL
354	ASP	236	LEU
366	VAL	237	ALA
413	THR	239	SER
415	PHE	242	ASN
416	THR	243	ILE
417	ASP	244	ILE
418	PHE	245	SER
419	VAL		
420	SER		
422	ASN		
425	ARG		

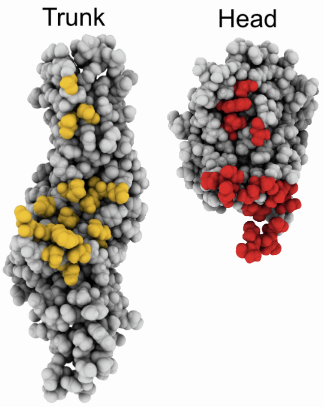

Figure S2. Characterization of TLPs purified in the absence of trypsin. (A) Maps of VP4 from *in situ* (top) and gradient purified (bottom) TLPs coloured by local resolution, with the corresponding FSC plots. (B) CsCl gradient purified TLPs. (C) SDS-PAGE gel of TLPs prepared in the presence of trypsin (left) and absence of trypsin (right). Molecular weight markers and the viral proteins are annotated. (D) Representative cryo-EM micrograph of TLPs gradient purified in the absence of trypsin. (E) The interacting residues participating in charge complementation and hydrophobic interactions on head and trunk domains of VP4-C are listed and depicted using a space-filling model. Related to Figure 3.

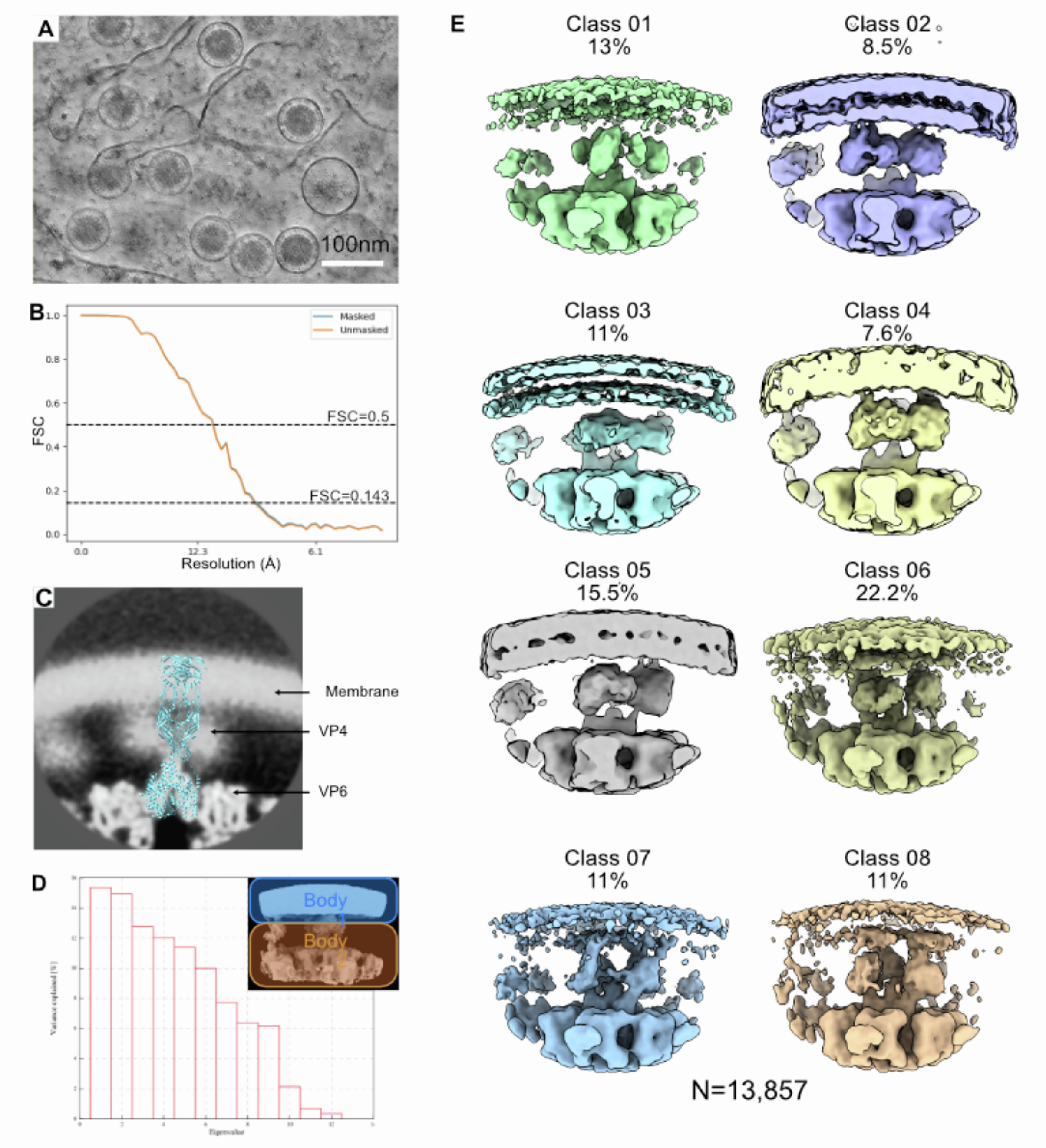

Figure S3 Characterization of pre-mature VP4. (A) A slice through a representative tomogram of rotavirus infected cells treated with thapsigargin. Scale bar 100nm. (B) Masked and unmasked FSC plot of the eDLP VP4. At the 0.143 cut-off the measured resolution is 8.5Å. (C) Central slice through the consensus average of the pre-mature VP4. Only the foot domain of the density is accounted for when the upright conformation of VP4 is docked into the density. (D) The percentage variance of each of the detected eigen-vectors is plotted. Inset describes the definition of the two bodies

used to perform multi-body analysis (see Methods). (E) Results of the 3D unaligned classification with the proportion of subtomograms in each class. The classification revealed an extra density adjacent to the trunk domain that is interpreted to be the head domain. Related to Figure 4.

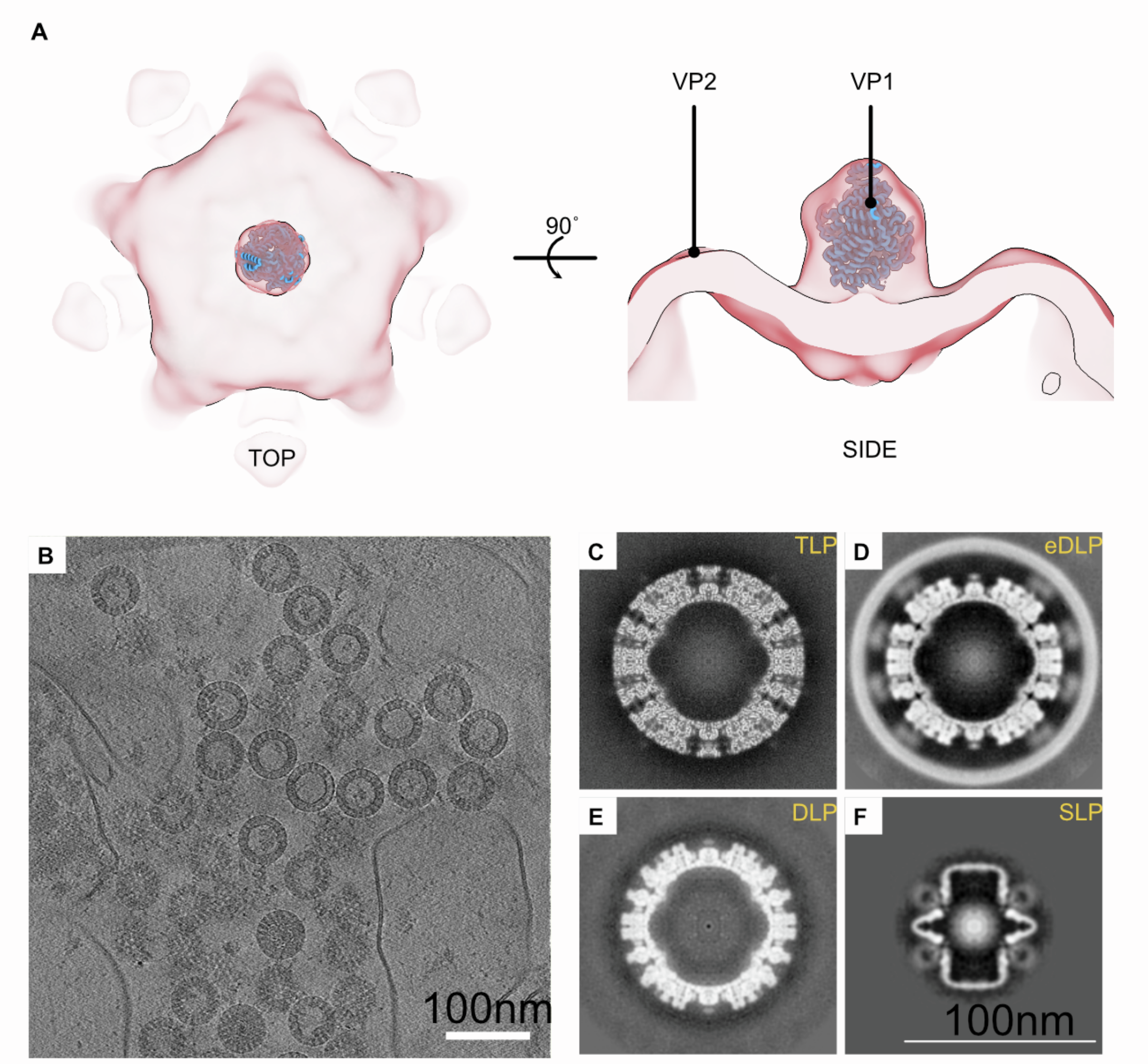

Figure S4. VP1 tsC mutant rotavirus is defective in genome packaging. (A) Size and shape similarity of the density present at the 5-fold vertices of the wt-SLP to the VP1 polymerase (blue). Left, top-down view. (Right) Side on view of a section offset from the 5-fold. (B) Section through a representative cryo-tomogram of a cell infected with the tsC mutant with most particles lacking the genome. Scale bar 100nm. (C-F) Central sections through STAs of the TLP, eDLP, DLP and SLP assembly intermediates of the tsC mutant. Related to Figure 5.

Microscope	Krios	
Voltage	300kV	
Collection Mode	Tilt series	Frame series
Detector	Falcon iV	K2 Summit
Energy filter	5eV	20eV
Number of Frames	8	40
Number of Tilts	41	-
Tilt range	"-40 to +40 in 2˚ increments"	-
Total Dose (e-/Å)	90.2	39.8
Defocus Range	1.5-3.5µm	0.5-5µm
Acquisition Scheme	Dose-symmetric	-
Pixel Size (Å/px)	1.97	0.82

Table S1. **Data collection parameters**. Related to Figure 2.

Condition	Wildtype				Thapsigargin	tsC		Untreated
**Particle Type**	**SLP**	**DLP**	**eDLP**	**TLP**	**eDLP**	**SLP**		**TLP**
**EMDB (icos/penton/spike)**	16772/-/-	16769/-/-	16771/16767/-	16773/16772/16774	-	-		(-/-/16146)
**PDB (penton/spike)**	-	-	-	8CO6/8COA	-	-		8BP8
**Number of Tomograms**	85				127	53	**Number of movies**	9461
Number of subtomograms*	28	42 (412)	138 (1557)	279 (1961)	1097 (65,820)	100	**Number of particles**	131944
Pixel size (Å/px)*	2.47 (1.97)					2.47	**Pixel size (Å/px)**	0.82
Map resolution (Å)*	26.9	12 (10.2)	7.7 (7.2)	6.9 (4.2)	5.82 (9.07)	19.1	**Map resolution (Å)**	2.74

Table S2. **Map resolutions and Particle numbers**. *Brackets indicate the relevant information for the sub-particles, related to Figure 2.

.

Refinement	**in situ TLP (spike)**	**in situ TlP (penton)**	**in vitro TLP (spike)**
R.M.S. Deviations			
Bond Length (Å)	0.004	0.003	0.004
Bond Angles(˚)	1.068	0.563	0.956
Validation			
Molprobity score	2.43	2.38	1.67
Poor rotamers (%)	0.26	3.91	0.28
Ramachandran plot			
Outliers (%)	0.02	0.02	0.02
Allowed (%)	8.66	5.52	3.41
Favoured (%)	91.32	94.45	96.57

Table S3. Refinement statistics, related to Figure 2.
